# Changes in immune parameters between pre-treatment and recurrence after (chemo) radiation therapy in patients with head and neck cancer

**DOI:** 10.1038/s41598-020-68938-8

**Published:** 2020-07-20

**Authors:** Takeharu Ono, Koichi Azuma, Akihiko Kawahara, Tatsuyuki Kakuma, Fumihiko Sato, Toshihiko Kawaguchi, Jun Akiba, Hirohito Umeno

**Affiliations:** 1 Department of Otolaryngology-Head and Neck Surgery, School of Medicine, Kurume University, Asahimachi 67, Kurume, Fukuoka 830-0011 Japan; 2 Division of Respirology, Neurology, and Rheumatology, Department of Internal Medicine, Kurume University School of Medicine, Kurume, Fukuoka Japan; 3 Department of Diagnostic Pathology, Kurume University Hospital, Kurume, Fukuoka Japan; 4 Biostatistics Center, Kurume University School of Medicine, Kurume, Fukuoka Japan

**Keywords:** Cancer, Immunology, Oncology

## Abstract

Squamous cell carcinoma of the head and neck (SCCHN) has a high recurrence rate after (chemo) radiation therapy [(C)RT]. The relationship between the changing levels of immune checkpoint molecules and immune cells in pre-(C)RT tissues and locally recurrent tissues in the irradiated field, after (C)RT completion, is not known. This study aimed to assess the changes in these immune parameters between pre-(C)RT tissue and the same area after local recurrence post-(C)RT. We retrospectively reviewed 30 (C)RT-treated patients with SCCHN. We performed immunohistochemical analyses on these immune parameters using paired tissue samples obtained pre-(C)RT and at local recurrence sites post-(C)RT. No significant changes in immune parameters were found between the pre-(C)RT and locally recurrent tissues. An increased density of CD8+ tumor-infiltrating lymphocytes (TILs) showed a significantly positive correlation with PD-L expression on tumor cells (TC-PD-L1). Patients with increased TC-PD-L1 expression and CD8+TIL density showed favourable prognosis, and one of them showed a favourable response to nivolumab therapy. Our study shows a positive association between TC-PD-L1 upregulation and increased CD8+TIL density, and demonstrates that patients with these changes have a favourable survival outcome.

## Introduction

Immune checkpoint inhibitors (ICI) are utilized in systemic therapies that induce T cells to specifically target and kill tumor cells^1^. Programmed death 1 (PD-1) is a receptor expressed on the T cell membrane, which binds to programmed death-ligand 1 (PD-L1) on tumor cells, resulting in T cell anergy and inhibition of antitumor activity^2^. In the case of squamous cell carcinoma of the head and neck (SCCHN), the “CheckMate 141” phase 3 trial demonstrated that nivolumab therapy, which inhibits the PD-l/PD-L1 and PD-L2 axis, increases the survival periods of people with recurrent/metastatic SCCHN (RMSCCHN) compared to the therapy selected by investigators^3^. The expression levels of PD-L1 on tumor and immune cells is a known predictive biomarker that correlates with the efficacy of PD-1/PD-L1 inhibitors^4^. This correlation was confirmed in a recent analysis of 2-year long-term survival data from CheckMate 141, showing that nivolumab therapy results in favourable overall survival (OS) and OS benefits in patients with PD-L1 expression ≥ 1%^4^.

Radiation therapy (RT) or chemo-radiation therapy (CRT) is now a standard procedure for patients with head and neck cancer^5^. Recent studies have shown that PD-L1 expression changed in response to chemotherapy (CT), RT, and molecular target therapy^6–8^. RT is known to induce cancer cell death and has positive immune-modulatory abilities, including causing the increased expression of MHC-class I molecules, releasing various tumor antigens, and accumulating tumor-infiltrating lymphocytes (TILs)^9–11^. Furthermore, a recent study showed that tumor cell PD-L1 expression is upregulated in response to IFN-gamma, produced by CD8+ T cells, through a process named adoptive immune resistance status. Thus, this kind of immune escape might indicate favourable responses to PD-1/PD-L1 inhibitors^12^.

Several studies have reported changes in the immune checkpoint molecule expression levels that correlate with immune cell levels between the tissue samples obtained at pre-treatment sites and sequentially surgically resected tissue after neoadjuvant CT or CRT^7,13,14^. Leduc et al.^15^ reported that docetaxel, platinum, and fluorouracil (TPF) induction chemotherapy for head and neck cancer increased PD-L1 expression on tumor cells and immune cells. Lim et al.^14^ reported that patients with oesophageal cancer receiving neoadjuvant chemo-radiotherapy displayed a significant increase in PD-L1 expression between pre-CRT and post-CRT evaluations, in 12 paired tissue samples.

However, to our knowledge, no information regarding the association of immune checkpoint molecules and immune cells between pre-(C)RT and local recurrence sites post-(C)RT is available. This study aimed to investigate the changes in immune parameters of paired-biopsy specimens obtained from pre-(C)RT and local recurrence tissue sites post-(C)RT in patients with head and neck cancer.

## Results

### Patient characteristics

The clinical characteristics of the 30 patients enrolled in this study are shown in Table 1. As for treatments before recurrence, 16 and 14 patients received cisplatin-based chemo-radiotherapy and radiotherapy, respectively. All patients were treated with external RT five times a week (1.8 or 2.0 Gy/fraction/day), using a three-dimensional (3D) method and a 4-megavolt X-ray beam linear accelerator. The patients received a total dosage and range of radiation of 66.0 Gy and 60–70.2 Gy, respectively. The median age of the patients at diagnosis of local recurrence was 66 years (range: 40–87 years), where 27 (90%) were male, and three were female (10%). The Eastern Cooperative Oncology Group (ECOG) performance status was 0 for all patients. The recurrence sites were the larynx, oropharynx, and hypopharynx in 18 (60%), 8 (27%), and 4 (13%) patients, respectively. The median follow-up time from tumor recurrence was 63.1 months (range 15.3–146.2). Regarding p-16 status, 6 (20%) and 24 (80%) patients showed positive and negative expression, respectively. Local recurrence statuses described as being of the early or advanced stage comprised 22 (73%) and 8 (27%) patients, respectively, and two patients (7%) had a regional recurrence. The mean, minimum, and maximum time elapsed between the treatment of the primary tumor and recurrence was 15.1, 1.4, and 47.4 months, respectively. Regarding treatment at recurrence, 22 (73%) patients received surgery, 3 (10%) received chemotherapy, 3 (10%) had cetuximab combined with chemotherapy, and 2 (7%) received nivolumab therapy.

**Table 1:** Patient characteristics (n = 30).

Variables	Total (%)
**Age**	
Mean	66
range	40–87
**Sex**	
Male	27 (90)
Female	3 (10)
**Recurrent site**	
Larynx	18 (60)
Oropharynx	8 (27)
hypopharynx	4 (13)
**Treatment before recurrence**	
Chemo-radiotherapy	16 (53)
Radiotherapy	14 (47)
**Radiation dosage before recurrence**	
Median	66.0 Gy
Range	60–70.2 Gy
**p16 status**	
Positive	6 (20)
Negative	24 (80)
**Local recurrence**	
Early	22 (73)
Advance	8 (27)
**Regional recurrence**	
Yes	2 (7)
No	28 (93)
**Treatment at recurrence**	
Surgery	22 (73)
Chemotherapy	3 (10)
Cetuximab + chemotherapy	3 (10)
Nivolumab	2 (7)
**Follow up (months) form recurrence**	
Median	63.1
Range	15.3–146.2

### Baseline immune parameters

The expression levels of PD-L1 on tumor cells (TC-PD-L1), PD-L1 on immune cells in the stroma (IC-PD-L1), PD-L2 on tumor cells (TC-PD-L2), PD-L2 on immune cells in the stroma (IC-PD-L2), HLA-class I expression, and CD8+TIL density, are shown in Table 1.

### Correlations between the changes in immune parameters before (C)RT and in local recurrences after (C)RT

**Figure 1:**
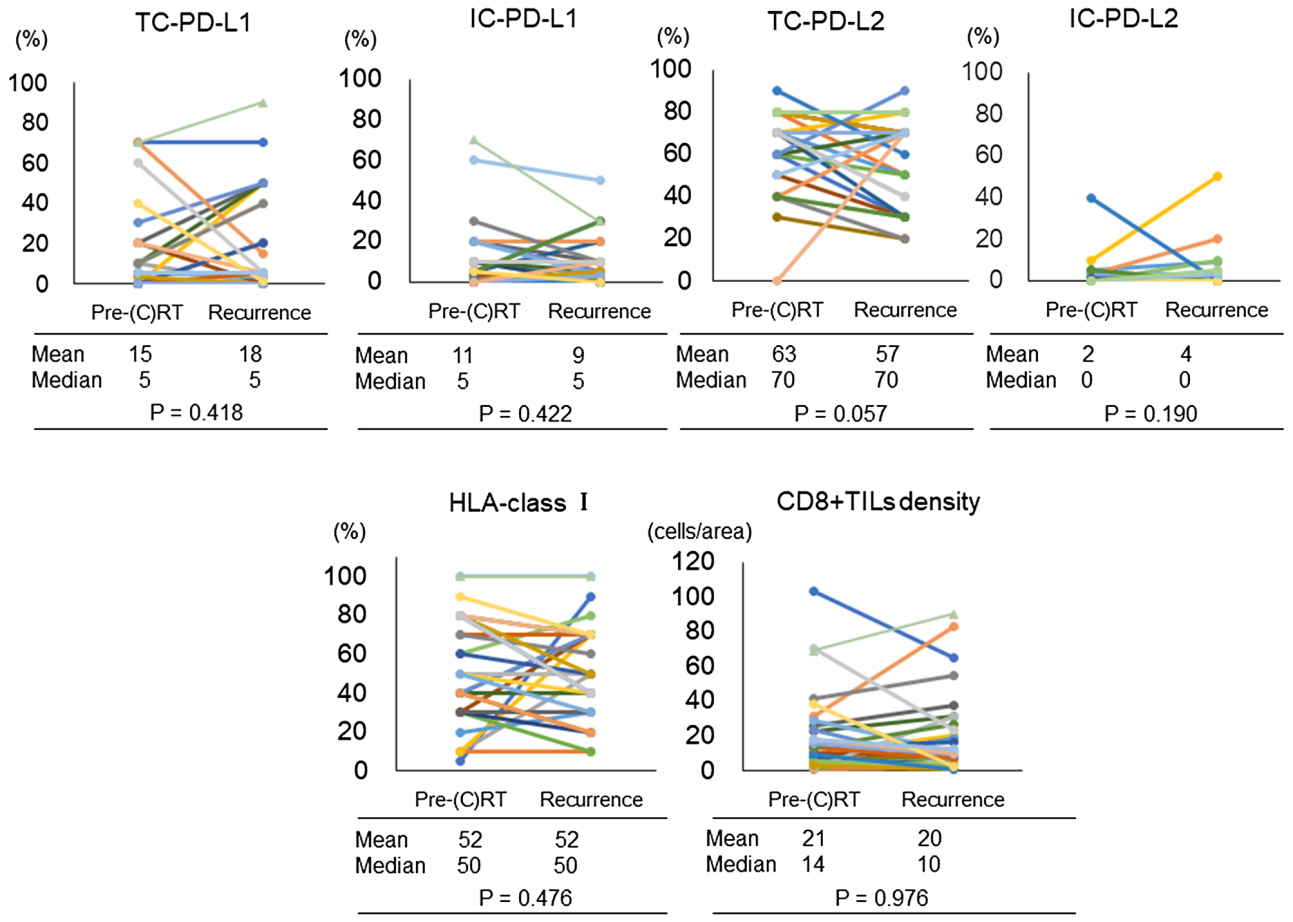
Changes to programmed death-ligand 1 expression on tumor cells (TC-PD-L1), PD-L1 on immune cells (IC-PD-L1), PD-L2 on tumor cells (TC-PD-L2), PD-L2 on immune cells (IC-PD-L2), HLA-class I, and CD8+TIL density before (C)RT and at local recurrence sites.

The correlations between changes in the density of TC-PD-L1, IC-PD-L1, TC-PD-L2, IC-PD-L2, HLA-class I, and CD8+TILs in pre-(C)RT and local recurrences in tissues are shown in Fig. 1. The mean expression levels of the markers in pre-(C)RT and locally recurrent tissue samples, respectively, were 15 and 18% for TC-PD-L1, 11 and 9% for IC-PD-L1, 63 and 57% for TC-PD-L2, 2 and 4% for IC-PD-L1, and 52 and 52% for HLA-class I. Additionally, these median expression levels in the pre-(C)RT and locally recurrent tissue samples showed no changes. The mean density level of the CD8+TILs in the pre-(C)RT and locally recurrent tissue samples was 21 and 20, respectively (median value: 14 and 10). There were no significantly definitive associations with the changes in these immune parameters (TC-PD-L1, *p* = 0.418; IC-PD-L1, *p* = 0.422; TC-PD-L2, *p* = 0.057; IC-PD-L2, *p* = 0.190; HLA-class I, *p* = 0.476; CD8+TILs, *p* = 0.976).

###  Correlations between changes in PD-L1 or PD-L2 expression on tumor cells, immune cells, or HLA-class I or CD8+TILs density in pre-(C)RT tissues and local recurrences after (C)RT

The correlations between changes in CD8+TIL density and TC-PD-L1, IC-PD-L1, TC-PD-L2, IC-PD-L2, or HLA-class I expression are shown in Table 2. Increases in CD8+TIL density showed a significant positive correlation with TC-PD-L1 expression (*p* = 0.007), whereas no significant differences were observed for IC-PD-L1 (*p* = 0.260), TC-PD-L2 (*p* = 1.000), IC-PD-L2 (*p* = 1.000), and HLA-class I expression (*p* = 0.226). The correlations between increased TC-PD-L1 expression and CD8+TIL density or with other changes in immune parameters (TC-PD-L1 expression and CD8+TIL density) and patient characteristics are shown in Supplementary Table S1. No significant correlations were found between increased TC-PD-L1 expression and CD8+TIL density or with other changes in immune parameters and patient characteristics.

**Table 2:** The correlations of change between CD8+ TIL density and immune parameters.

Immune parameters	CD8+ TILs density (%)		
	Increase	Decrease	*p *value
**TC-PD-L1 expression**			
Up	9 (64)	2 (13)	0.007*
NC or down	5 (36)	14 (87)	
**IC-PD-L1 expression**			
Up	3 (21)	7 (44)	0.260
NC or down	11 (79)	9 (56)	
**TC-PD-L2 expression**			
Up	3 (21)	4 (25)	1.000
NC or down	11 (79)	12 (75)	
**IC-PD-L2 expression**			
Up	5 (36)	5 (31)	1.000
NC or down	9 (64)	11 (69)	
**HLA-class I**			
Up	2 (14)	6 (38)	0.226
NC or down	12 (86)	10 (62)	

*NC* no change.

*p < 0.05.

### Survival analysis

**Figure 2:**
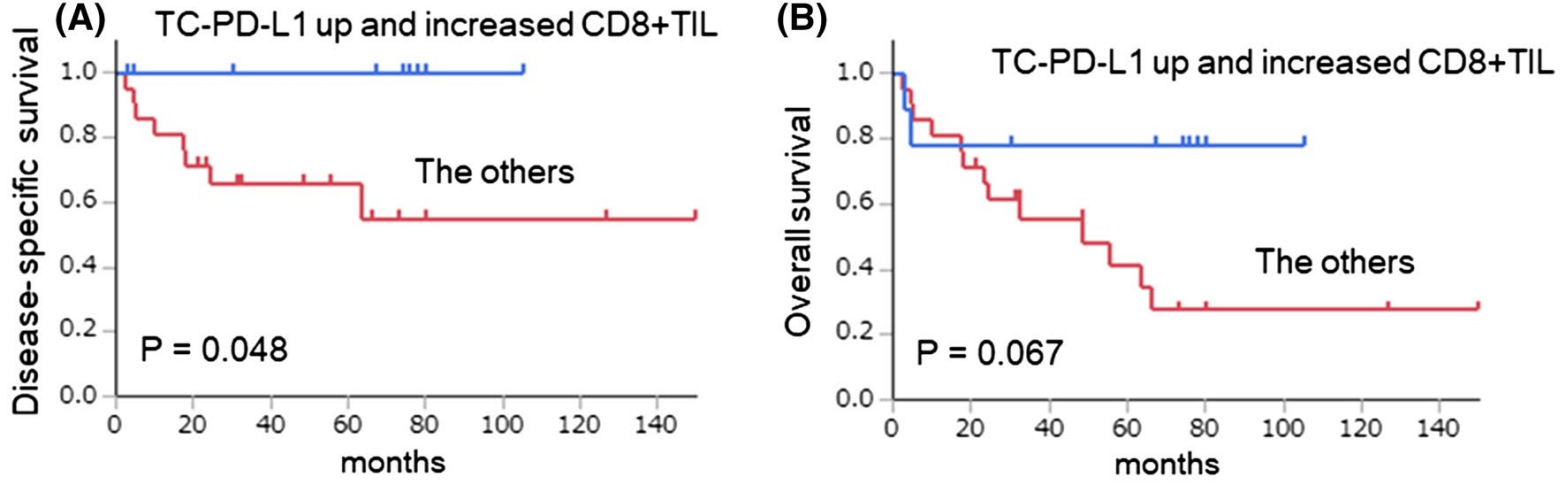
Kaplan–Meier analysis of disease-specific survival (**A**) and overall survival (**B**) for patients exhibiting both up-regulated TC-PD-L1 expression and increased CD8+TIL density or others. Significant differences were determined using a log-rank test.

A Kaplan–Meier analysis was performed to evaluate DSS and OS for the patients with both increased TC-PD-L1 expression and increased CD8+TIL density, as well as for patients in the other groups (patients with both decreased TC-PD-L1 and increased CD8+TIL or decreased CD8+TIL, and patients with both increased TC-PD-L1 and decreased CD8+TIL density) (Fig. 2). Patients with both increased TC-PD-L1 and increased CD8+TIL density indicated a favourable DSS (*p* = 0.048) and OS (*p* = 0.067).

### Response to nivolumab therapy

**Figure 3:**
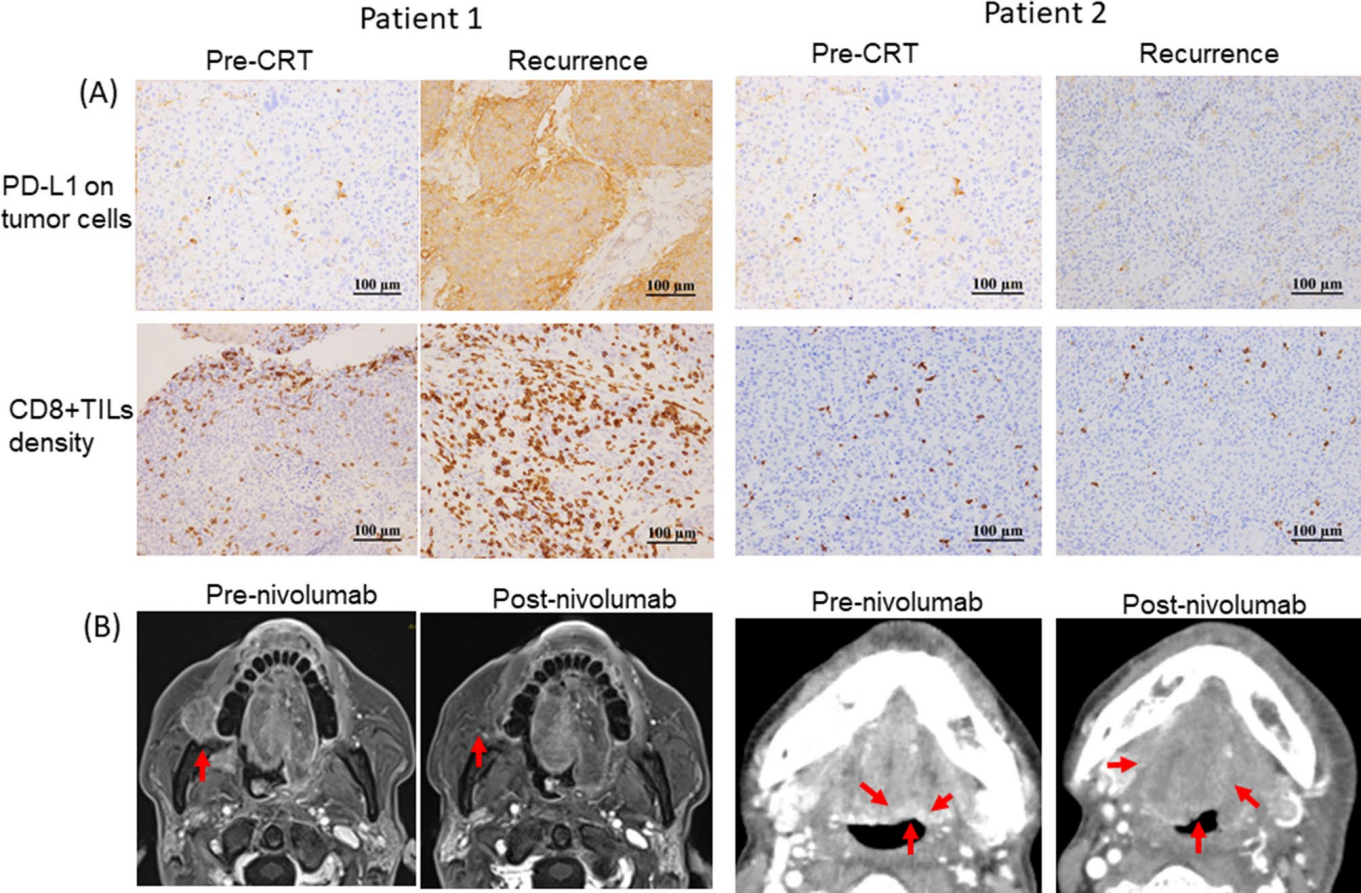
Histopathological features of TC-PD-L1 expression and CD8+TIL density in pre-(C)RT and recurrence tissues (**A**), and clinical images (Patient 1:MRI and Patient 2: CT) from the pre-and post-nivolumab therapy patients (**B**) are shown for patients 1 and 2, respectively.

Two patients, both of whom had p16-positive oropharyngeal cancer, received nivolumab therapy (Fig. 3). Patient 1, who received radiotherapy and concurrent cisplatin (total radiation dosage, 69.0 Gy), underwent tissue biopsy 3 months after the completion of the CRT, and experienced local recurrence, as evidenced by the biopsy. This patient, who had both increased TC-PD-L1 expression and increased CD8+TIL density, exhibited a complete response to the treatment after 4 cycles of nivolumab therapy. Patient 2, who received radiotherapy and concurrent cisplatin (total radiation dosage, 70.2 Gy), underwent tissue biopsy 4 months after the completion of the CRT, and experienced local recurrence, as confirmed by the biopsy. This patient, who did not have increased TC-PD-L1 expression and had decreased CD8 +TIL density, showed progressive tumor disease even after 4 cycles of nivolumab therapy.

## Discussion

A recent study reported that radiation-induced immune responses led to PD-L1 upregulation and increased dendritic and cytotoxic T cell activation and proliferation^16^. Herein, we investigated the association between changes in immune parameters, including PD-L1 using pre-(C)RT and locally recurrent paired tissue samples. Our results showed that there were no significant changes in the immune parameters between pre-(C)RT and local recurrence sites after (C)RT.

Recent reports have shown a definite increase in PD-L1 expression levels and immune infiltrating cells induced by chemotherapy and CRT. In the patient’s cohort with SCCHN, induction chemotherapy increased PD-L1 expression on tumor cells and immune cells, including CD8+TILs^15^. In studies involving patients with squamous oesophageal cancer or rectal cancer receiving CRT, CRT reportedly significantly increased PD-L1 expression^6,14^. Further, Dovedi et al.^17^ reported that in a pre-clinical study using a mouse model, radiation-induced upregulation of PD-L1 expression on tumor cells peaked at 72 h after RT and then began to decline. Additionally, Patel et al.^13^ reported that the analysis of PD-L1 expression at a median of 28 days after completion of RT might be suboptimal.

Accordingly, changes in immune parameters including PD-L1 expression, were closely associated with chemotherapy, (C)RT, and the period after the completion of (C)RT. Thus, we investigated this using recurrent tissue samples, but not pre-(C)RT tissue samples, to exactly evaluate the current tumor immune microenvironment (TIM). This is because of TIM between pre-(C)RT tumor tissue sites and tumor recurrence sites after (C)RT might differ. To the best of our knowledge, this is the first investigation of changes in immune parameters between pre-(C)RT and post-(C)RT recurrence in patients with head and neck cancer.

We examined the association of changes in immune checkpoint molecule expression and the number of immune cells and found a positive correlation between TC-PD-L1 expression and CD8+TIL density. Furthermore, patients with upregulated PD-L1 expression and an increase in CD8+TIL density had significantly more favourable prognoses. A recent report described the four types of TIM, type I (adaptive immune resistance), type II (immunological ignorance), type III (intrinsic induction), and type IV (tolerance)^18^. A TIM with both upregulated PD-L1 expression and an increased density of CD8+TILs was considered to be a type I TIM, in the conditions of immune escape induced by the inactivation of T cells^19^. Dovedi et al.^17^ found that PD-L1 expression in tumor cells induced by IFN-gamma, produced by CD8+T cells, may act as a biomarker for local antitumor response levels. Previous reports showed that, in several cancer types, type I TIMs in several cancer types had favourable prognoses compared to other types. In addition, type I TIM (adaptive immune resistance) may be the most likely to respond to PD-1/PD-L1 inhibitors^19–21^. Of the two patients undergoing nivolumab monotherapy, patient 1 had upregulated PD-L1 expression on tumor cells and an increased CD8+TIL density and showed a clinically complete response to nivolumab therapy. However, patient 2 showed no PD-L1 upregulation and no increase in CD8+TIL density, had a clinically defined progressive disease after nivolumab therapy, and died of cancer. Thus, these results suggest that investigating the changes in PD-L1 and CD8+TIL density might help evaluate an immunologic shift via interferon-gamma, induced by (C)RT; furthermore, this might be useful for predicting the responses to immune checkpoint inhibitor treatments in cases of recurrence after a definitive (C)RT.

Our study had several limitations that should be taken into consideration. First, this study was retrospective and consisted of a small number of patients. Second, clinical characteristics at the primary and treatment sites (CRT or RT) were heterogeneous. Third, precise evaluation of TIL densities, PD-L1, PD-L2, and HLA-class I expression might be affected by intratumoral heterogeneity, due to biopsy tissue specimens.

In summary, although we show no definite association between the changes in PD-L1 or PD-L2 expression (tumor and immune cells), TIL densities, and HLA-class I expression between pre-(C) RT tissue and the local recurrence sites after (C)RT, there was a positive association between the upregulation of PD-L1 expression on tumor cells and an increased CD8+TIL density. Furthermore, patients with both upregulation of PD-L1 expression on tumor cells and an increased CD8+TIL density had more favourable survival likelihoods compared with the other patients. Considering the limitations of this study, further large prospective studies are required to confirm our results.

## Methods

### Patients

We retrospectively screened 30 patients diagnosed with SCCHN at Kurume University Hospital between 2004 and 2016. The selected patients had been diagnosed pathologically with SCCHN, had local recurrence after the completion of radiotherapy or chemo-radiotherapy, and had adequate frozen histological specimens containing tumor cells in paired tissue samples from both pre-(C)RT and local recurrence sites. Local recurrence in the radiated field was regularly examined using trans-nasal endoscopy, and tissue biopsies were performed if local recurrence was suspected. The malignant positivity of the biopsy confirmed local recurrence. Patient exclusion criteria included those with distant metastases from the local recurrence, those undergoing palliative treatments, or those with inoperable disease. The present study was conducted in accordance with the provisions outlined in the Declaration of Helsinki and was approved by the Institutional Review Boards of Kurume university. Informed consent was obtained from the patients. However, if the patients died, informed consent was obtained from the patient’s family.

### Immunohistochemical (IHC) analysis

As previously reported^20,22^, we used 4-μm-thick sections of formalin-fixed, paraffin-embedded tissues. The tissue sections were mounted on glass slides and incubated with an anti-PD-L1 rabbit monoclonal antibody (clone D3; Cell Signaling Technology, Danvers, MA) using BenchMark ULTRA (Ventana Automated Systems, Inc., Tucson, AZ). Briefly, each slide was heat-treated using Ventana’s cell conditioning solution (CC1) for 30 min and incubated with the PD-L1 antibody for 30 min. The automated system used the ultraVIEW DAB detection kit with 3,3′ diaminobenzidine (DAB) as the chromogen. Mouse monoclonal human PD-L2 (1:200, Clone 176,611; R&D systems) antibody was used after treatment with proteinase K (Agilent/Dako, CA, USA) for 5 min, after which the antibody was added and incubated for 30 min^23^. This automated system used a refined polymer detection system (Leica Microsystems, Newcastle, UK) with HRP (Horseradish peroxidase)-polymer bound to an anti-mouse secondary antibody, using 3,3′ diaminobenzidine (DAB) as the chromogen. Immunostaining for HLA-class I (EMR8-5; ab70328; Abcam, Cambridge, England) and CD8 (Leica Microsystems, Newcastle-upon-Tyne, UK) was performed on the fully automated Bond-III system. Briefly, each slide was treated using the heat-induced epitope retrieval solution 2 for 10 min at 99 °C, before being incubated with the primary antibody for 30 min at room temperature. This was followed by incubation with the secondary antibody for 30 min at room temperature.

All IHC findings were evaluated by two experienced pathologists (A.K. and J.A.) who were unaware of the patients’ conditions. Any disagreement between the pathologists was resolved by a joint review to obtain a single consensus category. As previously reported^24^, the expression of PD-L1 (TC-PD-L1) and PD-L2 (TC-PD-L2) on tumor cells was evaluated using a defined tumor proportion score. The expression of PD-L1 (IC-PD-L1) and PD-L2 (IC-PD-L2) on immune cells (lymphocytes or macrophages) in the stroma occupying the tumor were assessed using the mononuclear immune cell density score. Furthermore, CD8+TILs were counted in five high-magnification fields of view (magnification: 40×), and these averages were assessed.

### Statistical analysis

Correlations between the changes to immune parameters in the local lesions before and after (C)RT were analyzed using a Wilcoxon signed-rank test. Disease-specific survival (DSS) and OS were calculated from the date of initial treatment after local recurrence to the date of death due to cancer or any cause, respectively. The Kaplan–Meier method was used to assess patient survival curves, and the log-rank test was used to evaluate the differences between groups. All tests were two-sided, and the differences were considered statistically significant at *p* < 0.05. Statistical analyses were performed using JMP Pro 14 statistical software (SAS Institute, Cary, NC).

## Supplementary information

Supplementary table S1.
