# Supplementary material for: Changes in immune parameters between pre-treatment and recurrence after (chemo) radiation therapy in patients with head and neck cancer
Source: Sci Rep. 2020 Jul 20;10:11973. doi: 10.1038/s41598-020-68938-8 (PMC7371733; doi:10.1038/s41598-020-68938-8)
Supplement: Supplementary file 1 — Supplementary table S1. [file 41598_2020_68938_MOESM1_ESM.docx]

**Changes in immune parameters between pre-treatment and recurrence after (chemo) radiation therapy in patients with head and neck cancer**

Takeharu Ono,^1^ Koichi Azuma,^2^ Akihiko Kawahara,^3^ Tatsuyuki Kakuma,^4^ Fumihiko Sato,^1^ Toshihiko Kawaguchi,^1^ Jun Akiba,^3^ Hirohito Umeno,^1^

^1^Department of Otolaryngology- Head and Neck Surgery, Kurume University School of Medicine, Kurume, Fukuoka, Japan

^2^Division of Respirology, Neurology, and Rheumatology, Department of Internal Medicine, Kurume University School of Medicine, Kurume, Fukuoka, Japan.

^3^Department of Diagnostic Pathology, Kurume University Hospital, Kurume, Fukuoka, Japan.

^4^Biostatistics Center, Kurume University School of Medicine, Kurume, Fukuoka, Japan.

**Supplementary Table 1**. Characteristics in patients with increased TC-PD-L1 and CD8+TILs or the others.

|  | Increased TC-PD-L1 and CD8+TILs group (%) | The others group (%) | p-value^a^ |
| --- | --- | --- | --- |
| Age  　≤ 66  > 66 | 5 (56)  4 (44) | 11 (52)  10 (48) | 1.000 |
| Sex  Male  Female | 0  9 (100) | 18 (86)  3 (14) | 0.535 |
| Recurrent site  Larynx  Oropharynx  hypopharynx | 5 (56)  3 (33)  1 (11) | 13 (62)  5 (24)  3 (14) | 0.855 |
| Treatment before recurrence  Chemo-radiotherapy  Radiotherapy | 6 (67)  3 (33) | 10 (48)  11 (52) | 0.440 |
| p16 status  Positive  Negative | 3 (33)  6 (67) | 3 (14)  18 (86) | 0.329 |
| Local recurrence  Early  Advanced | 6 (67)  3 (33) | 16 (76)  5 (24) | 0.667 |
| Regional recurrence  Yes  No | 1 (11)  8 (89) | 1 (5)  20 (95) | 0.434 |
| Treatment at recurrence  Surgery  Chemotherapy or  cetuximab+chemotherapy  Nivolumab | 6 (67)  2 (22)  1 (11) | 16 (76)  4 (19)  1 (5) | 0.821 |

^a^Fisher’s exact test.
